# Supplementary material for: Differential Response of MDA-MB-231 and MCF-7 Breast Cancer Cells to In Vitro Inhibition with CTLA-4 and PD-1 through Cancer-Immune Cells Modified Interactions
Source: Cells. 2021 Aug 10;10(8):2044. doi: 10.3390/cells10082044 (PMC8392578; doi:10.3390/cells10082044)
Supplement: Supplementary file 1 [file cells-10-02044-s001.zip › cells-1229919-supplementary.pdf]

Title: Differential response of MDA-MB-231 and MCF-7 breast cancer cells to *in vitro* inhibition with CTLA-4 and PD-1 through cancer-immune cells modified interactions.

Running Title: CTLA-4 and PD-1 inhibition-related effects on differential modulation of immune responses against breast cancer cells.

Authors: Kamil Grubczak[1,*], Anna Kretowska-Grunwald[1], Dawid Groth[1], Izabela Poplawska[3], Andrzej Eljaszewicz[1], Lukasz Bolkun[4], Aleksandra Starosz[1], Jordan M. Holl[1], Marta Mysliwiec[5], Joanna Kruszewska[5], Marek Z. Wojtukiewicz[5], Marcin Moniuszko[1,2,*]

[1] - Department of Regenerative Medicine and Immune Regulation, Medical University of Bialystok, JerzegoWaszyngtona 13, 15-269 Bialystok, Poland

[2] – Department of Allergology and Internal Medicine, Medical University of Bialystok, M. Sklodowskiej-Curie 24A, 15-276 Bialystok

[3] - Department of Medical Pathomorphology, Medical University of Bialystok, Jerzego Waszyngtona 13, 15-269 Bialystok, Poland

[4] – Department of Haematology, Medical University of Bialystok, M. Sklodowskiej-Curie 24A, 15-276 Bialystok

[5] - Department of Oncology, Medical University of Bialystok, Ogrodowa 12, 15-027 Bialystok, Poland

Corresponding Authors (*):

Kamil Grubczak, PhD and Marcin Moniuszko, MD, PhD; mailing address: Department of Regenerative Medicine and Immune Regulation, Medical University of Bialystok, Jerzego Waszyngtona 13, 15-269 Bialystok, Poland; phone: +85 748 59 72; fax: +85 748 59 71; email: kamil.grubczak@umb.edu.pl; marcin.moniuszko@umb.edu.pl

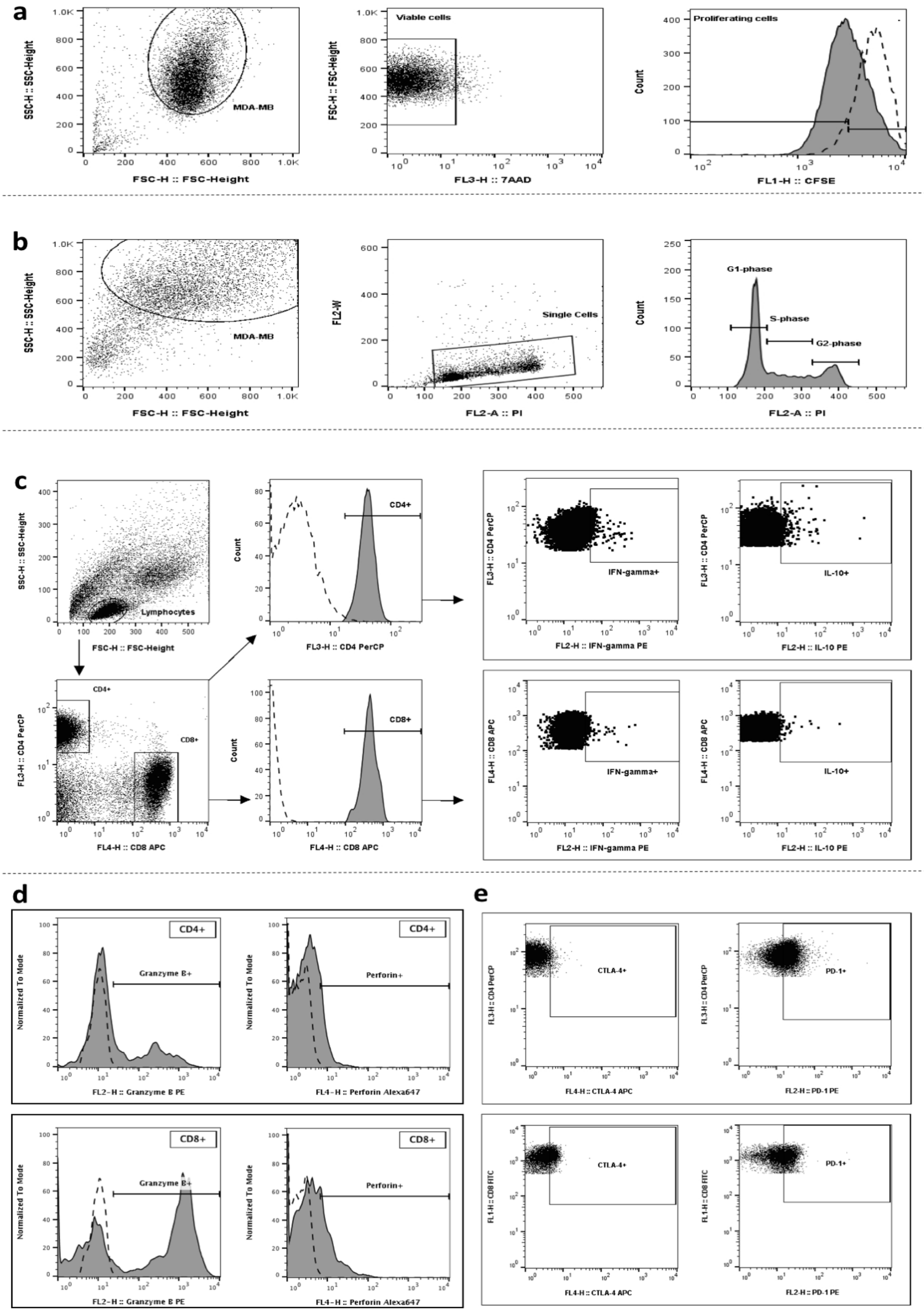

Figure S1. Gating strategy in flow cytometric analysis for assessment of MDA-MB-231 and MCF-7 breast cancer cell proliferation (a) and cell cycle status (b), and evaluation of IFN-gamma and IL-10 production (c), perforin and granzyme B production (d) or CTLA-4 and PD-1 expression (e) within lymphocytes.

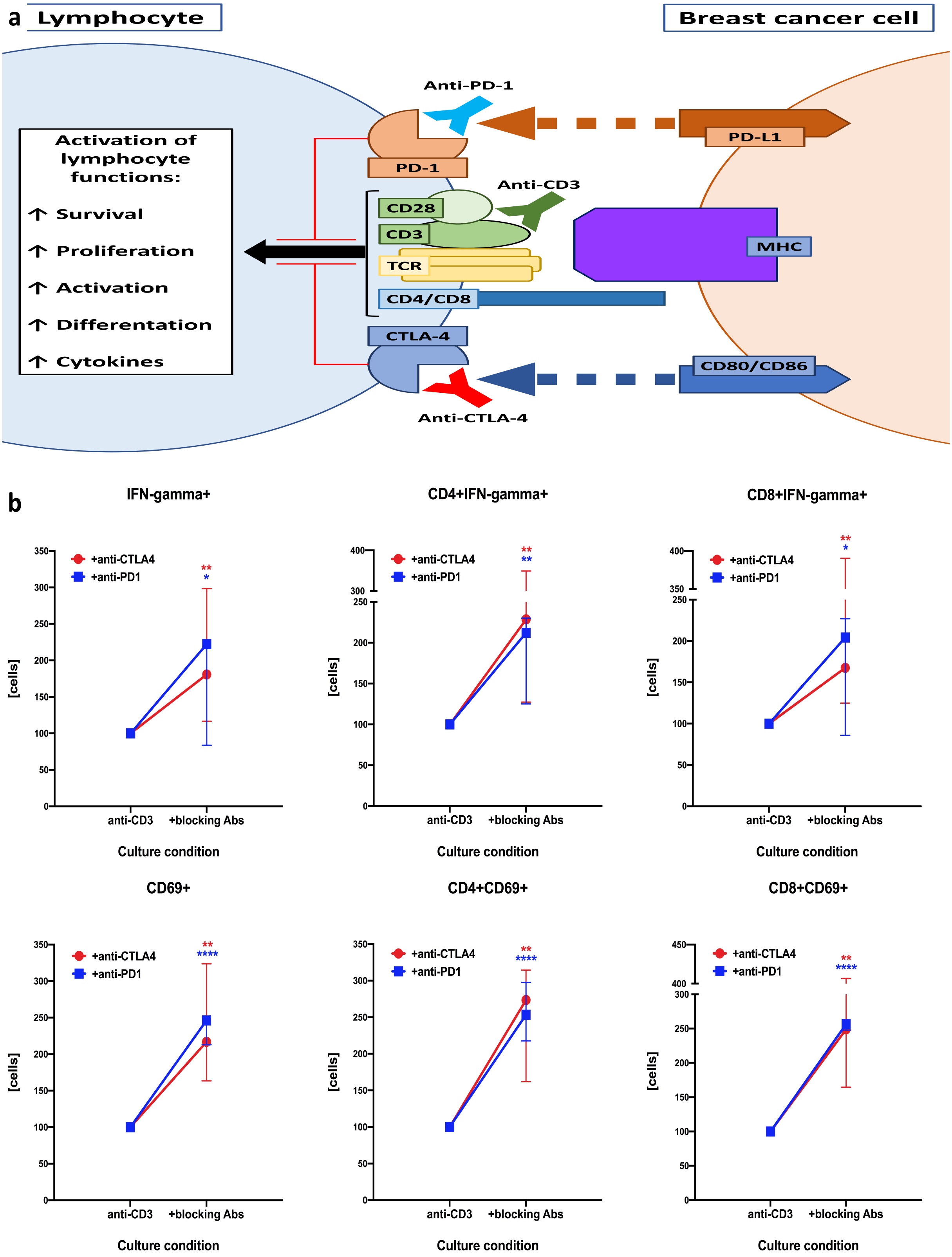

Figure S 2. Assessment of anti-CTLA-4 and anti-PD-1 blocking antibodies. Schematic presentation of anti-CTLA-4 and anti-PD-1 application influence on prevention from CD80/CD86- or PD-L1-induced inhibition of lymphocytes activation – induced by anti-CD3 stimulation (a). Flow cytometric confirmation of enhanced lymphocytes activation in anti-CTLA-4 or anti-PD-1 blocking compared to cells stimulated with anti-CD3 only (b).

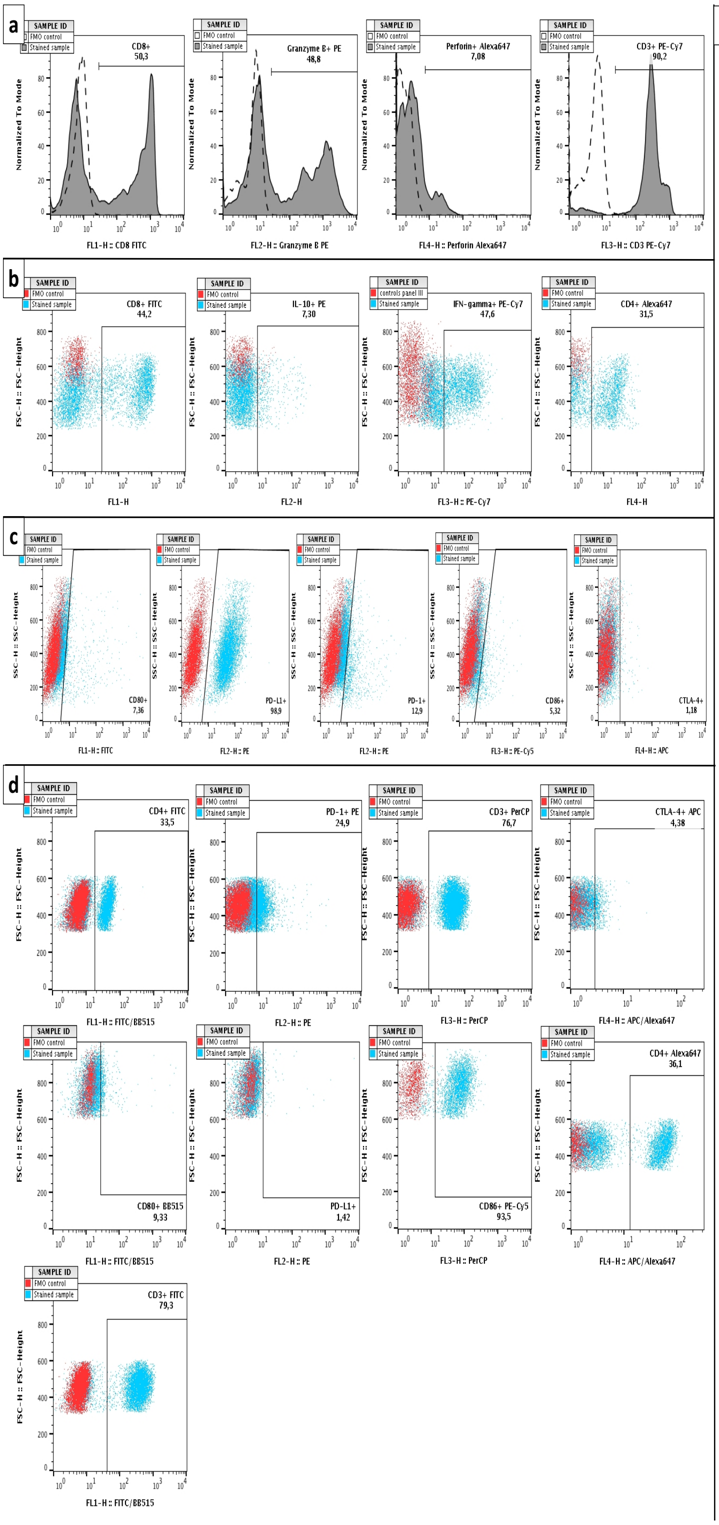

e

Antibodies used in flow cytometric analyses

Target	Reactivity	Clone	Host	Fluorochrome
CD3	human	SK7	mouse	FITC
CD3	human	SK7	mouse	PE-Cy7
CD3	human	SK7	mouse	PerCP
CD4	human	RPA-T4	mouse	FITC
CD4	human	RPA-T4	mouse	AlexaFluo647
CD4	human	SK3	mouse	PerCP
CD8	human	HIT8a	mouse	FITC
CD8	human	SK1	mouse	APC
CD80	human	L307.4	mouse	BB515
CD86	human	IT2.2	mouse	PE-Cy5
CD152/CTLA-4	human	BNI3	mouse	APC
CD274/PD-L1	human	MIH1	mouse	PE
CD279/PD-1	human	MIH4	mouse	PE
Granzyme B	human	GB11	mouse	PE
IFN-gamma	human	4S.B3	mouse	PE
IL-10	human	JES3-19F1	rat	PE
Perforin	human	δG9	mouse	AlexaFluo647

Figure S3. Confirmation of the monoclonal antibodies specificity and reactivity used in flow cytometric analyses. Flow cytometric data, histograms and dot plots, demonstrating implemented antibodies efficacy in monitoring studied parameters including: cytokines production within lymphocytes (a and b), selected immune checkpoint proteins in MDA-MB-231/MCF-7 (c) and lymphocytes (PBMC) (d). Additional complete list of antibodies with exxential features prepared on the basis of information provided by the company (BD Bioscience) (e).
